# 2-Methyl-4-oxopentan-2-aminium 2-sulfamoylbenzoate

**DOI:** 10.1107/S1600536809027007

**Published:** 2009-07-18

**Authors:** Muhammad Rafique, Ghulam Hussain, Waseeq Ahmad Siddiqui, M. Nawaz Tahir

**Affiliations:** a Department of Chemistry, University of Sargodha, Sargodha, Pakistan; b Department of Physics, University of Sargodha, Sargodha, Pakistan

## Abstract

In the title salt, C_6_H_14_NO^+^·C_7_H_6_NO_4_S^−^, the 2-sulfamoylbenzoate anion has two intra­molecular hydrogen bonds, forming a five membered C—H⋯O and a seven-membered N—H⋯O twisted ring with ring motifs *S*(5) and *S*(7), respectively, while the 2-methyl-4-oxopentan-2-aminium cation also has a stabilizing intra­molecular N—H⋯O hydrogen bond with a twisted *S*(6) ring motif. The anions form inversion-related dimers with *R*_2_^2^(8) ring motifs through inter­molecular N—H⋯O hydrogen bonding. The dimers and cations are further linked and stabilized through inter­molecular N—H⋯O and C—H⋯O bonds, forming zigzag-shaped layers that extend along the crystallographic *a* axis.

## Related literature

For related structures, see: Akram *et al.* (2008 ▶); Schmidt *et al.* (1997 ▶); Siddiqui *et al.* (2007 ▶). For the definition of hydrogen-bond patterns used for graph-set analysis, see: Bernstein *et al.* (1995 ▶). For applications of aldol condensation, see: Afonso & Crespo (2005 ▶).
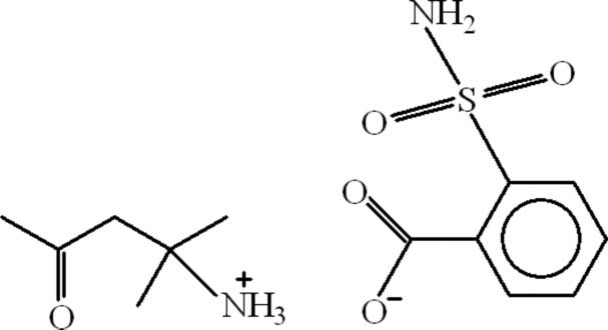


## Experimental

### 

#### Crystal data

C_6_H_14_NO^+^·C_7_H_6_NO_4_S^−^
*M* *_r_* = 316.37
Orthorhombic, 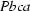

*a* = 21.1917 (9) Å
*b* = 6.3897 (2) Å
*c* = 23.3751 (11) Å
*V* = 3165.2 (2) Å^3^
*Z* = 8
Mo *K*α radiation
μ = 0.23 mm^−1^
*T* = 296 K
0.28 × 0.10 × 0.08 mm

#### Data collection

Bruker Kappa APEXII CCD diffractometer
Absorption correction: multi-scan (*SADABS*; Bruker, 2005 ▶) *T*_min_ = 0.975, *T*_max_ = 0.983
32870 measured reflections
4111 independent reflections
2961 reflections with *I* > 2σ(*I*)
*R*_int_ = 0.032

#### Refinement

*R*[*F* ^2^ > 2σ(*F* ^2^)] = 0.037
*wR*(*F* ^2^) = 0.110
*S* = 1.04
4111 reflections
200 parameters
H atoms treated by a mixture of independent and constrained refinement
Δρ_max_ = 0.32 e Å^−3^
Δρ_min_ = −0.29 e Å^−3^

### 

Data collection: *APEX2* (Bruker, 2007 ▶); cell refinement: *SAINT* (Bruker, 2007 ▶); data reduction: *SAINT*; program(s) used to solve structure: *SHELXS97* (Sheldrick, 2008 ▶); program(s) used to refine structure: *SHELXL97* (Sheldrick, 2008 ▶); molecular graphics: *ORTEP-3 for Windows* (Farrugia, 1997 ▶) and *PLATON* (Spek, 2009 ▶); software used to prepare material for publication: *WinGX* (Farrugia, 1999 ▶) and *PLATON*.

## Supplementary Material

Crystal structure: contains datablocks global, I. DOI: 10.1107/S1600536809027007/si2187sup1.cif

Structure factors: contains datablocks I. DOI: 10.1107/S1600536809027007/si2187Isup2.hkl

Additional supplementary materials:  crystallographic information; 3D view; checkCIF report

## Figures and Tables

**Table 1:** Hydrogen-bond geometry (Å, °)

*D*—H⋯*A*	*D*—H	H⋯*A*	*D*⋯*A*	*D*—H⋯*A*
N1—H1*A*⋯O4^i^	0.80 (2)	2.21 (2)	3.008 (2)	172 (2)
N1—H1*B*⋯O2	0.85 (2)	2.10 (2)	2.848 (2)	145.4 (18)
N2—H2*A*⋯O1	0.89	1.94	2.8124 (18)	167
N2—H2*B*⋯O2^ii^	0.89	1.87	2.7622 (18)	176
N2—H2*C*⋯O5	0.89	2.18	2.824 (2)	129
N2—H2*C*⋯O1^iii^	0.89	2.50	2.9878 (17)	115
C3—H3⋯O4	0.93	2.47	2.870 (2)	106
C6—H6⋯O2^iii^	0.93	2.52	3.444 (2)	170

Symmetry codes: (i) 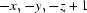; (ii) 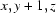; (iii) 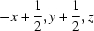.

**Fig. 1.**
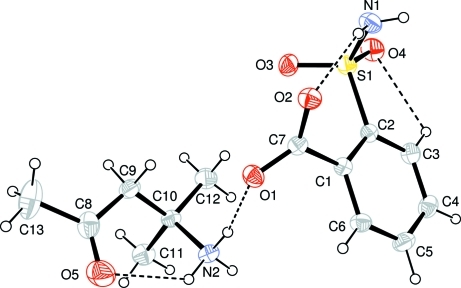
View of the title compound with the atom numbering scheme. The thermal ellipsoids are drawn at the 30% probability level. H-atoms are shown by small circles of arbitrary radii. Intramolecular H-bonds are shown by dotted lines.

**Fig. 2.**
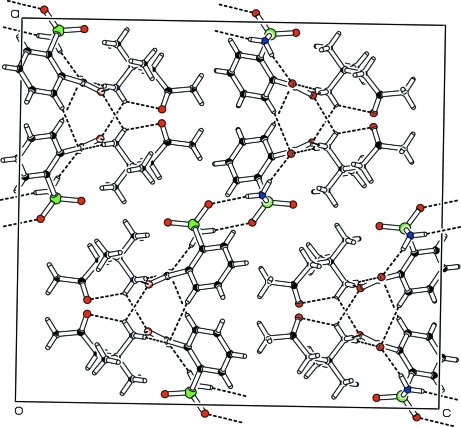
The partial packing (PLATON; Spek, 2009) which shows that molecules form dimers of 2-sulfamoylbenzoate and connected to 2-methyl-4-oxopentan-2-aminium through intermolecular H-bondings.

## supplementary crystallographic information

### Comment

We reported the crystal structure of Tetraaquabis(2-sulfamoylbenzoato) manganese(II) (Akram *et al.*, 2008). In continuation of synthesizing metal complexes of *o*-sulfamoylbenzoic acid (Siddiqui *et al.*, 2007), the title compound (I), (Fig. 1) is prepared in a try of tin complex. The crystal structure of bis(2-Methyl-4-oxopent-2-yl)ammonium bis(phthalato) -beryllium(I) (Schmidt *et al.*, 1997) contains the cation, 2-Methyl-4-oxopentan-2-aminium, of (I). The title compound is an example of aldol condensation which is routinely applied to prepare products used in the fields of biological sciences, industrial catalysis and green chemistry (Afonso & Crespo, 2005).

In the title compound, there are two moieties. In the anion, 2-sulfamoylbenzoate, two intramolecular H-bonds form five and seven membered twisted rings [S(5) and S(7)], while the cation, 2-methyl-4-oxopentan-2-aminium, has also an intramolecular H-bonding with a twisted ring S(6) (Bernstein *et al.*, 1995) (Fig. 1). The cations and anions are connected with each other through H-bonding of the N—H···O type. The 2-sulfamoylbenzoate form dimers with R_2_^2^(8) ring motifs through intermolecular H-bonding of type N1—H1A···O4^i^ (Table 1) [symmetry code: i = -*x*, -*y*, -*z* + 1]. The dimers are linked to 2-methyl-4-oxopentan-2-aminium moietes through intermolecular H-bondings, (Table 1, Fig. 2), forming zigzag shaped layers that extend along the crystallographic *a*-axis.

### Experimental

A suspension of (1.0 g, 4.97 mmol) o-sulfamoylbenzoic acid (Siddiqui *et al.*, 2007), tin(II) chloride dihydrate (0.561 g, 2.49 mmol) and sodium carbonate (0.3 g, 2.83 mmol) was subjected to reflux in a mixture of acetone, methanol, water (1:1:1) for 4 h. The volume of the reaction mixture was reduced to half on a rotary evaporator (11 torr) at room temperature and its pH was adjusted to 12, using aqueous ammonia solution and left over night at the same temperature. The white product was filtered, washed with cold distilled water and dried at room temperature. The product was recrystallized at 313 K from aqueous methanol to obtain colorless needle shaped crystals.

### Refinement

The coordinates of H-atoms of NH_2_ group were refined. The H-atoms were positioned geometrically, with N—H = 0.89 for NH_3_, C—H = 0.93, 0.96 and 0.97 Å for aryl, methyl and ethylenic H, respectively and constrained to ride on their parent atoms, with U_iso_(H) = xU_eq_(C, N), where x = 1.2 for all H atoms.

### Crystal data

**Table:**

C_6_H_14_NO^+^·C_7_H_6_NO_4_S^−^	*F*(000) = 1344
*M**_r_* = 316.37	*D*_x_ = 1.328 Mg m^−^^3^
Orthorhombic, *P**b**c**a*	Mo *K*α radiation, λ = 0.71073 Å
Hall symbol: -P 2ac 2ab	Cell parameters from 4111 reflections
*a* = 21.1917 (9) Å	θ = 2.6–28.8°
*b* = 6.3897 (2) Å	µ = 0.23 mm^−^^1^
*c* = 23.3751 (11) Å	*T* = 296 K
*V* = 3165.2 (2) Å^3^	Needle, colorless
*Z* = 8	0.28 × 0.10 × 0.08 mm

### Data collection

**Table:**

Bruker Kappa APEXII CCD diffractometer	4111 independent reflections
Radiation source: fine-focus sealed tube	2961 reflections with *I* > 2σ(*I*)
graphite	*R*_int_ = 0.032
Detector resolution: 7.40 pixels mm^-1^	θ_max_ = 28.8°, θ_min_ = 2.6°
ω scans	*h* = −27→28
Absorption correction: multi-scan (*SADABS*; Bruker, 2005)	*k* = −8→5
*T*_min_ = 0.975, *T*_max_ = 0.983	*l* = −31→30
32870 measured reflections	

### Refinement

**Table:**

Refinement on *F*^2^	Primary atom site location: structure-invariant direct methods
Least-squares matrix: full	Secondary atom site location: difference Fourier map
*R*[*F*^2^ > 2σ(*F*^2^)] = 0.037	Hydrogen site location: inferred from neighbouring sites
*wR*(*F*^2^) = 0.110	H atoms treated by a mixture of independent and constrained refinement
*S* = 1.04	*w* = 1/[σ^2^(*F*_o_^2^) + (0.0482*P*)^2^ + 1.1665*P*] where *P* = (*F*_o_^2^ + 2*F*_c_^2^)/3
4111 reflections	(Δ/σ)_max_ < 0.001
200 parameters	Δρ_max_ = 0.32 e Å^−^^3^
0 restraints	Δρ_min_ = −0.29 e Å^−^^3^

### Special details

**Table:**

Geometry. Bond distances, angles etc. have been calculated using the rounded fractional coordinates. All su's are estimated from the variances of the (full) variance-covariance matrix. The cell esds are taken into account in the estimation of distances, angles and torsion angles
Refinement. Refinement of *F*^2^ against ALL reflections. The weighted *R*-factor *wR* and goodness of fit *S* are based on *F*^2^, conventional *R*-factors *R* are based on *F*, with *F* set to zero for negative *F*^2^. The threshold expression of *F*^2^ > σ(*F*^2^) is used only for calculating *R*-factors(gt) *etc*. and is not relevant to the choice of reflections for refinement. *R*-factors based on *F*^2^ are statistically about twice as large as those based on *F*, and *R*- factors based on ALL data will be even larger.

### Fractional atomic coordinates and isotropic or equivalent isotropic displacement parameters (Å^2^)

**Table:**

	*x*	*y*	*z*	*U*_iso_*/*U*_eq_	
O5	0.23168 (8)	0.6184 (3)	0.16582 (7)	0.0740 (6)	
N2	0.17797 (6)	0.6721 (2)	0.27542 (6)	0.0367 (4)	
C8	0.18387 (11)	0.5283 (3)	0.15179 (8)	0.0534 (6)	
C9	0.12913 (9)	0.5023 (3)	0.19207 (8)	0.0451 (5)	
C10	0.12064 (7)	0.6719 (3)	0.23730 (7)	0.0368 (5)	
C11	0.11386 (9)	0.8877 (3)	0.21123 (9)	0.0504 (6)	
C12	0.06400 (8)	0.6216 (3)	0.27479 (9)	0.0523 (6)	
C13	0.17685 (15)	0.4323 (5)	0.09405 (10)	0.0902 (10)	
S1	0.03137 (2)	0.12048 (7)	0.41313 (2)	0.0366 (1)	
O1	0.18858 (5)	0.25236 (19)	0.31033 (5)	0.0426 (3)	
O2	0.15851 (5)	−0.02972 (17)	0.35914 (5)	0.0413 (4)	
O3	0.02610 (5)	0.1446 (2)	0.35274 (5)	0.0482 (4)	
O4	−0.02263 (5)	0.1683 (2)	0.44799 (6)	0.0519 (4)	
N1	0.04974 (8)	−0.1189 (2)	0.42641 (8)	0.0464 (5)	
C1	0.15011 (7)	0.2984 (2)	0.40450 (6)	0.0307 (4)	
C2	0.09432 (7)	0.2818 (2)	0.43663 (6)	0.0317 (4)	
C3	0.08632 (8)	0.3954 (3)	0.48632 (7)	0.0408 (5)	
C4	0.13337 (9)	0.5284 (3)	0.50512 (8)	0.0465 (6)	
C5	0.18748 (9)	0.5514 (3)	0.47338 (8)	0.0473 (6)	
C6	0.19563 (8)	0.4383 (3)	0.42347 (7)	0.0405 (5)	
C7	0.16605 (7)	0.1638 (2)	0.35297 (7)	0.0324 (4)	
H2A	0.18196	0.54745	0.29202	0.0441*	
H2B	0.17365	0.77014	0.30218	0.0441*	
H2C	0.21218	0.69889	0.25458	0.0441*	
H9A	0.13377	0.36904	0.21148	0.0540*	
H9B	0.09076	0.49517	0.16952	0.0540*	
H11A	0.15160	0.92237	0.19057	0.0605*	
H11B	0.10717	0.98862	0.24105	0.0605*	
H11C	0.07852	0.88900	0.18554	0.0605*	
H12A	0.06955	0.48618	0.29186	0.0628*	
H12B	0.02641	0.62158	0.25188	0.0628*	
H12C	0.06029	0.72529	0.30432	0.0628*	
H13A	0.21496	0.45282	0.07257	0.1083*	
H13B	0.14222	0.49701	0.07436	0.1083*	
H13C	0.16884	0.28511	0.09803	0.1083*	
H1A	0.0454 (10)	−0.141 (3)	0.4599 (10)	0.0557*	
H1B	0.0843 (11)	−0.148 (3)	0.4094 (9)	0.0557*	
H3	0.04917	0.38246	0.50721	0.0490*	
H4	0.12842	0.60205	0.53914	0.0558*	
H5	0.21875	0.64337	0.48550	0.0568*	
H6	0.23233	0.45630	0.40221	0.0486*	

### Atomic displacement parameters (Å^2^)

**Table:**

	*U*^11^	*U*^22^	*U*^33^	*U*^12^	*U*^13^	*U*^23^
O5	0.0674 (10)	0.0762 (11)	0.0783 (11)	−0.0084 (8)	0.0259 (8)	−0.0127 (9)
N2	0.0321 (6)	0.0351 (7)	0.0430 (8)	−0.0012 (5)	−0.0019 (6)	−0.0067 (6)
C8	0.0666 (13)	0.0476 (10)	0.0461 (10)	0.0138 (10)	0.0033 (9)	−0.0028 (9)
C9	0.0520 (10)	0.0389 (8)	0.0443 (10)	−0.0064 (8)	−0.0092 (8)	−0.0041 (7)
C10	0.0326 (8)	0.0361 (8)	0.0417 (9)	−0.0027 (6)	−0.0045 (7)	−0.0009 (7)
C11	0.0517 (10)	0.0403 (9)	0.0593 (12)	0.0047 (8)	−0.0053 (9)	0.0025 (9)
C12	0.0348 (9)	0.0671 (12)	0.0550 (11)	−0.0065 (8)	−0.0003 (8)	0.0020 (10)
C13	0.110 (2)	0.110 (2)	0.0506 (13)	0.0401 (18)	−0.0048 (13)	−0.0204 (14)
S1	0.0269 (2)	0.0441 (2)	0.0389 (2)	−0.0018 (2)	0.0009 (2)	0.0023 (2)
O1	0.0429 (6)	0.0449 (6)	0.0401 (6)	0.0007 (5)	0.0096 (5)	−0.0001 (5)
O2	0.0435 (6)	0.0334 (6)	0.0469 (7)	0.0033 (5)	0.0055 (5)	−0.0053 (5)
O3	0.0396 (6)	0.0637 (8)	0.0412 (7)	−0.0052 (6)	−0.0085 (5)	0.0042 (6)
O4	0.0308 (6)	0.0665 (8)	0.0583 (8)	0.0012 (5)	0.0095 (5)	0.0013 (7)
N1	0.0480 (8)	0.0423 (8)	0.0489 (9)	−0.0057 (7)	0.0092 (7)	0.0034 (7)
C1	0.0286 (7)	0.0301 (7)	0.0335 (8)	0.0032 (5)	−0.0002 (6)	0.0007 (6)
C2	0.0293 (7)	0.0327 (7)	0.0332 (8)	0.0033 (6)	−0.0012 (6)	0.0027 (6)
C3	0.0397 (8)	0.0444 (9)	0.0383 (9)	0.0057 (7)	0.0050 (7)	−0.0009 (7)
C4	0.0569 (11)	0.0444 (9)	0.0382 (9)	0.0048 (8)	−0.0033 (8)	−0.0103 (8)
C5	0.0493 (10)	0.0416 (9)	0.0511 (10)	−0.0069 (8)	−0.0102 (8)	−0.0071 (8)
C6	0.0348 (8)	0.0415 (8)	0.0452 (10)	−0.0051 (7)	0.0017 (7)	−0.0012 (7)
C7	0.0230 (6)	0.0369 (8)	0.0373 (8)	0.0027 (6)	0.0001 (6)	−0.0021 (6)

### Geometric parameters (Å, °)

**Table:**

S1—O4	1.4377 (13)	C11—H11B	0.9600
S1—N1	1.6086 (14)	C11—H11C	0.9600
S1—C2	1.7731 (15)	C11—H11A	0.9600
S1—O3	1.4244 (13)	C12—H12A	0.9600
O5—C8	1.211 (3)	C12—H12C	0.9600
O1—C7	1.2416 (19)	C12—H12B	0.9600
O2—C7	1.2551 (17)	C13—H13A	0.9600
N2—C10	1.507 (2)	C13—H13B	0.9600
N2—H2B	0.8900	C13—H13C	0.9600
N2—H2C	0.8900	C1—C7	1.518 (2)
N2—H2A	0.8900	C1—C2	1.405 (2)
N1—H1B	0.85 (2)	C1—C6	1.388 (2)
N1—H1A	0.80 (2)	C2—C3	1.380 (2)
C8—C9	1.503 (3)	C3—C4	1.382 (3)
C8—C13	1.490 (3)	C4—C5	1.374 (3)
C9—C10	1.525 (3)	C5—C6	1.383 (3)
C10—C11	1.514 (3)	C3—H3	0.9300
C10—C12	1.521 (2)	C4—H4	0.9300
C9—H9A	0.9700	C5—H5	0.9300
C9—H9B	0.9700	C6—H6	0.9300
			
S1···O2	3.1262 (12)	H1A···O4^iv^	2.21 (2)
O1···N2	2.8124 (18)	H1A···C4^ii^	3.01 (2)
O1···N2^i^	2.9878 (17)	H1B···O2	2.10 (2)
O2···N2^ii^	2.7622 (18)	H1B···C7	2.95 (2)
O2···S1	3.1262 (12)	H2A···O1	1.9400
O2···N1	2.848 (2)	H2A···H12A	2.4100
O2···O3	3.0227 (15)	H2A···C7	2.8600
O3···C7	2.9683 (18)	H2A···H9A	2.4300
O3···O2	3.0227 (15)	H2B···O2^vii^	1.8700
O4···C4^iii^	3.235 (2)	H2B···H11B	2.4400
O4···N1^iv^	3.008 (2)	H2B···H12C	2.4200
O5···C11	3.213 (3)	H2B···C7^vii^	2.7900
O5···C13^v^	3.255 (3)	H2C···O1^v^	2.5000
O5···C8^v^	3.189 (3)	H2C···H11A	2.4300
O5···N2	2.824 (2)	H2C···O5	2.1800
O1···H9A	2.6900	H2C···C8	2.7100
O1···H2C^i^	2.5000	H3···H3^iii^	2.5900
O1···H2A	1.9400	H3···O4	2.4700
O1···H6	2.6800	H4···O4^iii^	2.7000
O1···H11B^ii^	2.9000	H5···C6^v^	2.9900
O2···H2B^ii^	1.8700	H5···H13A^ix^	2.5500
O2···H1B	2.10 (2)	H6···C7^v^	2.7800
O2···H12C^ii^	2.9000	H6···O1	2.6800
O2···H6^i^	2.5200	H6···O2^v^	2.5200
O3···H12B^vi^	2.6900	H9A···O1	2.6900
O3···H11C^vi^	2.8600	H9A···H2A	2.4300
O3···H9B^vi^	2.7000	H9A···H11B^ii^	2.5900
O3···H12A	2.7600	H9A···H12A	2.4400
O4···H13B^vi^	2.8100	H9B···H13B	2.4800
O4···H3	2.4700	H9B···O3^x^	2.7000
O4···H4^iii^	2.7000	H9B···H11C	2.5600
O4···H1A^iv^	2.21 (2)	H9B···H12B	2.4900
O5···H11A	2.6400	H11A···O5	2.6400
O5···H2C	2.1800	H11A···H2C	2.4300
O5···H11A^i^	2.8300	H11A···O5^v^	2.8300
O5···H13C^v^	2.8400	H11A···C8	2.7600
N1···O2	2.848 (2)	H11B···H9A^vii^	2.5900
N1···C4^ii^	3.407 (2)	H11B···H12C	2.4500
N1···O4^iv^	3.008 (2)	H11B···O1^vii^	2.9000
N2···O1	2.8124 (18)	H11B···H2B	2.4400
N2···O1^v^	2.9878 (17)	H11C···H9B	2.5600
N2···O2^vii^	2.7622 (18)	H11C···H12B	2.5600
N2···O5	2.824 (2)	H11C···O3^x^	2.8600
C4···N1^vii^	3.407 (2)	H12A···H9A	2.4400
C4···O4^iii^	3.235 (2)	H12A···H2A	2.4100
C7···O3	2.9683 (18)	H12A···O3	2.7600
C8···O5^i^	3.189 (3)	H12B···H9B	2.4900
C11···O5	3.213 (3)	H12B···O3^x^	2.6900
C13···O5^i^	3.255 (3)	H12B···H11C	2.5600
C4···H13C^viii^	3.0500	H12C···O2^vii^	2.9000
C4···H1A^vii^	3.01 (2)	H12C···H2B	2.4200
C6···H5^i^	2.9900	H12C···H11B	2.4500
C7···H1B	2.95 (2)	H13A···H5^xi^	2.5500
C7···H2B^ii^	2.7900	H13B···H9B	2.4800
C7···H2A	2.8600	H13B···O4^x^	2.8100
C7···H6^i^	2.7800	H13C···O5^i^	2.8400
C8···H2C	2.7100	H13C···C4^xii^	3.0500
C8···H11A	2.7600		
			
O3—S1—C2	107.65 (7)	C10—C11—H11B	109.00
O3—S1—O4	118.44 (7)	H12A—C12—H12C	109.00
O3—S1—N1	108.25 (9)	H12A—C12—H12B	109.00
N1—S1—C2	108.12 (8)	C10—C12—H12C	109.00
O4—S1—N1	106.58 (9)	H12B—C12—H12C	109.00
O4—S1—C2	107.44 (7)	C10—C12—H12A	109.00
H2B—N2—H2C	109.00	C10—C12—H12B	109.00
H2A—N2—H2C	109.00	C8—C13—H13A	109.00
H2A—N2—H2B	109.00	H13A—C13—H13B	109.00
C10—N2—H2A	109.00	C8—C13—H13B	109.00
C10—N2—H2B	109.00	C8—C13—H13C	109.00
C10—N2—H2C	109.00	H13B—C13—H13C	109.00
H1A—N1—H1B	121 (2)	H13A—C13—H13C	109.00
S1—N1—H1B	109.0 (13)	C6—C1—C7	117.63 (13)
S1—N1—H1A	109.2 (14)	C2—C1—C7	124.66 (12)
C9—C8—C13	116.4 (2)	C2—C1—C6	117.57 (13)
O5—C8—C9	121.92 (18)	S1—C2—C1	120.76 (10)
O5—C8—C13	121.7 (2)	S1—C2—C3	118.30 (12)
C8—C9—C10	116.54 (16)	C1—C2—C3	120.91 (14)
N2—C10—C9	108.40 (13)	C2—C3—C4	120.16 (16)
C9—C10—C12	110.03 (15)	C3—C4—C5	119.76 (17)
C11—C10—C12	110.46 (15)	C4—C5—C6	120.26 (17)
N2—C10—C11	108.28 (14)	C1—C6—C5	121.28 (16)
N2—C10—C12	107.21 (13)	O1—C7—O2	126.17 (15)
C9—C10—C11	112.29 (15)	O1—C7—C1	117.67 (12)
C8—C9—H9B	108.00	O2—C7—C1	116.02 (13)
C8—C9—H9A	108.00	C2—C3—H3	120.00
H9A—C9—H9B	107.00	C4—C3—H3	120.00
C10—C9—H9A	108.00	C3—C4—H4	120.00
C10—C9—H9B	108.00	C5—C4—H4	120.00
C10—C11—H11C	109.00	C4—C5—H5	120.00
H11B—C11—H11C	109.00	C6—C5—H5	120.00
H11A—C11—H11B	109.00	C1—C6—H6	119.00
H11A—C11—H11C	109.00	C5—C6—H6	119.00
C10—C11—H11A	109.00		
			
O4—S1—C2—C1	−168.62 (11)	C7—C1—C2—S1	−8.69 (19)
O4—S1—C2—C3	9.43 (15)	C7—C1—C2—C3	173.31 (14)
N1—S1—C2—C1	76.71 (14)	C2—C1—C6—C5	2.4 (2)
N1—S1—C2—C3	−105.24 (14)	C7—C1—C6—C5	−173.44 (16)
O3—S1—C2—C1	−40.02 (13)	C2—C1—C7—O1	135.91 (15)
O3—S1—C2—C3	138.03 (13)	C2—C1—C7—O2	−48.2 (2)
O5—C8—C9—C10	29.1 (3)	C6—C1—C7—O1	−48.5 (2)
C13—C8—C9—C10	−152.4 (2)	C6—C1—C7—O2	127.34 (15)
C8—C9—C10—C12	−179.37 (16)	S1—C2—C3—C4	−177.83 (14)
C8—C9—C10—N2	−62.4 (2)	C1—C2—C3—C4	0.2 (2)
C8—C9—C10—C11	57.2 (2)	C2—C3—C4—C5	1.7 (3)
C6—C1—C2—S1	175.74 (12)	C3—C4—C5—C6	−1.5 (3)
C6—C1—C2—C3	−2.3 (2)	C4—C5—C6—C1	−0.6 (3)

Symmetry codes: (i) −*x*+1/2, *y*−1/2, *z*; (ii) *x*, *y*−1, *z*; (iii) −*x*, −*y*+1, −*z*+1; (iv) −*x*, −*y*, −*z*+1; (v) −*x*+1/2, *y*+1/2, *z*; (vi) −*x*, *y*−1/2, −*z*+1/2; (vii) *x*, *y*+1, *z*; (viii) *x*, −*y*+1/2, *z*+1/2; (ix) −*x*+1/2, −*y*+1, *z*+1/2; (x) −*x*, *y*+1/2, −*z*+1/2; (xi) −*x*+1/2, −*y*+1, *z*−1/2; (xii) *x*, −*y*+1/2, *z*−1/2.

### Hydrogen-bond geometry (Å, °)

**Table:**

*D*—H···*A*	*D*—H	H···*A*	*D*···*A*	*D*—H···*A*
N1—H1A···O4^iv^	0.80 (2)	2.21 (2)	3.008 (2)	172 (2)
N1—H1B···O2	0.85 (2)	2.10 (2)	2.848 (2)	145.4 (18)
N2—H2A···O1	0.89	1.94	2.8124 (18)	167
N2—H2B···O2^vii^	0.89	1.87	2.7622 (18)	176
N2—H2C···O5	0.89	2.18	2.824 (2)	129
N2—H2C···O1^v^	0.89	2.50	2.9878 (17)	115
C3—H3···O4	0.93	2.47	2.870 (2)	106
C6—H6···O2^v^	0.93	2.52	3.444 (2)	170

Symmetry codes: (iv) −*x*, −*y*, −*z*+1; (vii) *x*, *y*+1, *z*; (v) −*x*+1/2, *y*+1/2, *z*.
